# Metformin-NSAIDs Molecular Salts: A Path towards Enhanced Oral Bioavailability and Stability

**DOI:** 10.3390/pharmaceutics15020449

**Published:** 2023-01-29

**Authors:** Francisco Javier Acebedo-Martínez, Alicia Domínguez-Martín, Carolina Alarcón-Payer, Carolina Garcés-Bastida, Cristóbal Verdugo-Escamilla, Jaime Gómez-Morales, Duane Choquesillo-Lazarte

**Affiliations:** 1 Laboratorio de Estudios Cristalográficos, IACT, CSIC-Universidad de Granada, Avda. de las Palmeras 4, 18100 Armilla, Spain; 2 Department of Inorganic Chemistry, Faculty of Pharmacy, University of Granada, 18071 Granada, Spain; 3 Servicio de Farmacia, Hospital Universitario Virgen de las Nieves, 18014 Granada, Spain

**Keywords:** metformin, NSAIDs, molecular salts, crystal engineering, mechanochemistry

## Abstract

According to the World Health Organization, more than 422 million people worldwide have diabetes. The most common oral treatment for type 2 diabetes is the drug metformin (MTF), which is usually formulated as a hydrochloride to achieve higher water solubility. However, this drug is also highly hygroscopic, thus showing stability problems. Another kind of worldwide prescribed drug is the non-steroidal anti-inflammatory drug (NSAID). These latter, on the contrary, show a low solubility profile; therefore, they must be administered at high doses, which increases the probability of secondary effects. In this work, novel drug-drug pharmaceutical solids combining MTF-NSAIDs have been synthesized in solution or by mechanochemical methods. The aim of this concomitant treatment is to improve the physicochemical properties of the parent active pharmaceutical ingredients. After a careful solid-state characterization along with solubility and stability studies, it can be concluded that the new molecular salt formulations enhance not only the stability of MTF but also the solubility of NSAIDs, thus giving promising results regarding the development of these novel pharmaceutical multicomponent solids.

## 1. Introduction

According to the World Health Organization (WHO), diabetes is defined as a “chronic, metabolic disease characterized by elevated levels of blood glucose” [1]. WHO estimates as 422 million the number of worldwide adult patients diagnosed with diabetes, and this number rises to 537 million according to the International Diabetes Federation [2]. In 2045, the number of patients with diabetes is believed to reach 784 million, therefore being diabetes a real challenge for healthcare systems [1,2]. There are two types of diabetes. Type 1 diabetes, once so-called juvenile diabetes, occurs when the pancreas does not produce insulin, whereas type 2 diabetes occurs when the body becomes resistant to insulin, and it most often develops in adults. Nonetheless, the prevalence of overweight individuals and obesity in the younger age populations, along with insufficient physical activity, is directly related to the increasing occurrence of type 2 diabetes in younger populations, too [1,3].

**Scheme 1:**
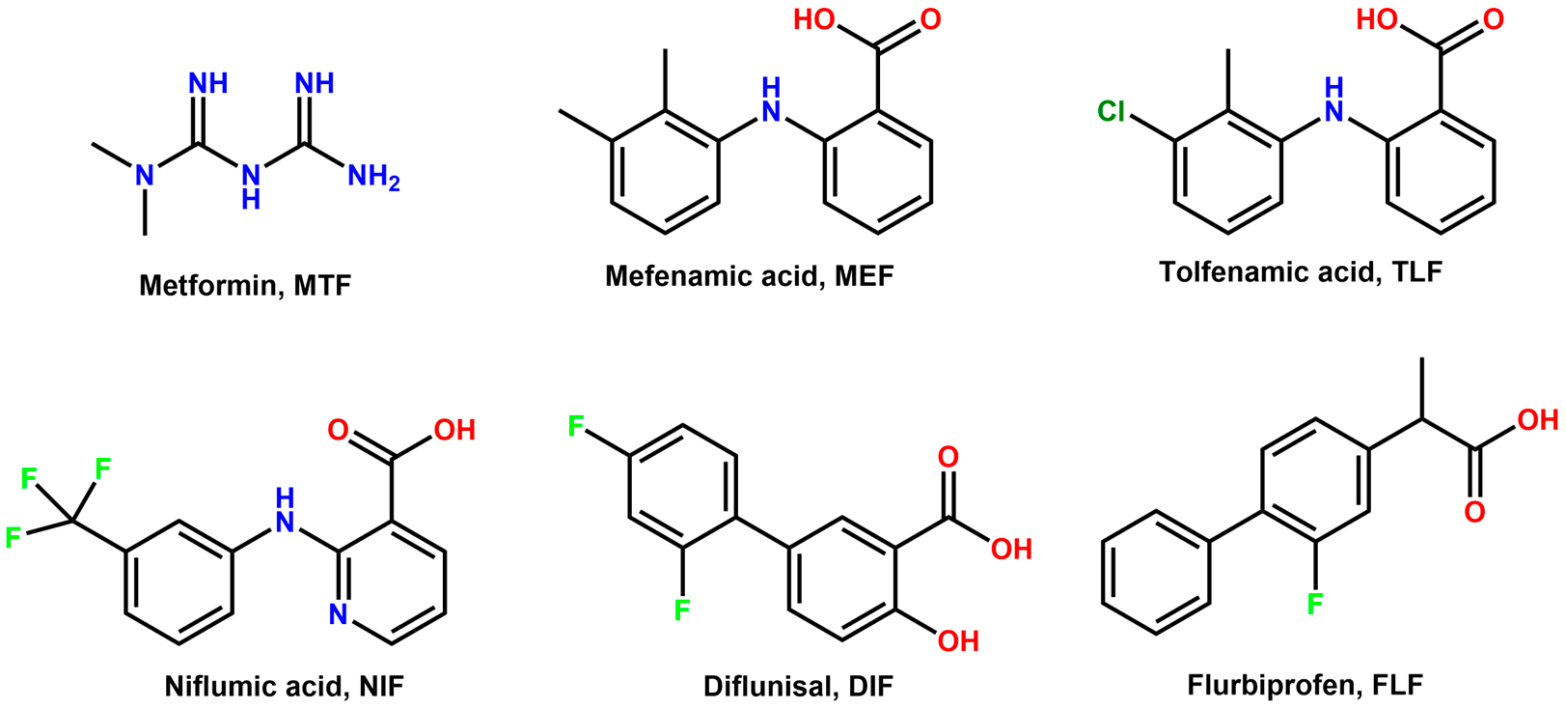
Chemical formula of metformin (MTF), mefenamic acid (MEF), tolfenamic acid (TLF), niflumic acid (NIF), diflunisal (DIF), and flurbiprofen (FLP).

Metformin (MTF) is a biguanide antihyperglycemic agent (Scheme 1) used as a first-line pharmacological treatment in the management of type 2 diabetes due to its efficacy, safety profile, and low cost for patients [4,5]. MTF exerts its antihyperglycemic action by increasing insulin sensitivity in peripheral tissues and reducing hepatic gluconeogenesis [6]. It is most commonly prescribed alone as metformin hydrochloride (MTF·HCl), i.e., chemically defined as a salt, in order to increase solubility and stability [7,8,9]. Although MTF·HCl solubility is outstanding [10], the low gastrointestinal permeability of MTF [11] eventually leads to gastrointestinal disorders in about 20–30% of chronic patients due to accumulation in the enterocytes within the small intestine [12,13]. Regarding the molecular stability of metformin, the instability profile and degradation products are detailed in its official Pharmacopeia monograph [14]. In addition, several stability assessments on aqueous media agree on metformin’s instability in alkaline solutions and its being less sensitive to oxidants [15,16]. Unfortunately, glycemic control with MTF in type 2 diabetes is still a challenge that concerns physicians due to its complexity [17]. Hence, its prescription, along with other antidiabetic drugs, is not rare in clinics.

Non-steroidal anti-inflammatory drugs (NSAIDs) work to relieve pain, reduce inflammation, and bring down fever [18], thus being one of the most commonly prescribed medications worldwide. Despite their chemical diversity, they are generally poorly soluble [18], which is an important drawback of this kind of drug. This fact requires an increase in their dosage, increasing the probability of side effects and drug interactions. Therefore, enhancing the solubility of NSAIDs is also quite an active research area within the pharmaceutical industry.

Diabetic neuropathy is a serious diabetes complication in which overall peripheral nerves are affected, whose symptoms include pain and numbness in extremities [19]. Thereby the concomitant administration of MTF and NSAIDs is very frequent [20], even if NSAIDs are not considered especially effective at treating neuropathic pain unless there is inflammation [19]. Despite the latter, their massive use is a reality in clinics, most likely due to: (i) the lack of non-opioid alternatives in the treatment of mild-moderate pain, (ii) the ‘over the counter’ (OTC) character of several NSAIDs, and (iii) their safety profile. Even if they are generally regarded as safe, a rare, although serious, condition known as lactic acidosis has been reported for long-term MTF treatments connected to acute renal failure [21,22]. On the other hand, NSAIDs are known for their nephrotoxicity [23]. Therefore, patients with concomitant and chronic treatments of MTF and NSAIDs are more susceptible to developing renal impairment [24].

In this work, crystal engineering tools have been used to yield drug–drug multicomponent pharmaceutical solids. This novel approach is rather interesting for the pharmaceutical industry since it allows for the improvement of the physicochemical properties of parent active pharmaceutical ingredients (APIs) without modifying their chemical structure, thus being a relatively inexpensive method compared to the development of a complete drug pipeline [25]. The proposed novel formulations involve the antidiabetic drug metformin (MTF) and five different NSAIDs, i.e., Niflumic acid (NIF), Diflunisal (DIF), Mefenamic acid (MEF), Tolfenamic acid (TLF), and Flurbiprofen (FLF) (Scheme 1).

The aim of the study is to explore novel treatment alternatives that might increase the oral bioavailability of the aforementioned drugs via the formation of multidrug salts, as well as reduce the side effects associated with this combined therapy. To this purpose, five (MTF)(NSAIDs) salts have been synthesized. Structure-physicochemical properties relationships of the new multicomponent pharmaceutical solids have been assessed thanks to a comprehensive solid-state analysis of the crystallographic structures. Moreover, solubility and stability have been evaluated in the new formulations and compared to the isolated parent APIs, showing promising results.

## 2. Materials and Methods

### 2.1. Materials

All APIs and solvents used in this work were purchased from commercial sources and used as received. Metfomin·HCl, tolfenamic acid, niflumic acid, and flurbiprofen were obtained from TCI Europe (Zwijndrecht, Belgium). Diflunisal and mefenamic acid were obtained from Sigma-Aldrich (St. Louis, CA, USA). Ethyl acetate HPLC grade and absolute ethanol were obtained from labkem (Barcelona, Spain).

### 2.2. Salt Syntheses

#### 2.2.1. Metformin Hydrochloride Neutralization

To obtain the MTF base, MTF·HCl was neutralized by stirring 10 mmol of MTF·HCl (1.656 g) and 10 mmol of NaOH (0.4 g) in 60 mL of isopropanol at room temperature, using a magnetic stirrer and a sealed glass beaker to avoid evaporation. After 24 h, the solution was filtered using 0.22 µm syringe filters to remove the NaCl (insoluble in isopropanol). The solvent of the clear filtered obtained (containing the MTF base) was removed using a rotatory evaporator set at 50 °C and 30 rpm. The remaining powder was dried and characterized by powder X-ray diffraction (PXRD) to confirm the purity of the MTF base obtained.

#### 2.2.2. Mechanochemical Synthesis

The mechanochemical synthesis of MTF salts was conducted using Liquid-Assisted Grinding (LAG) in a Retsh MM2000 ball mill (Retsch, Haan, Germany), operating at a 25-Hz frequency, using stainless steel jars along with stainless steel balls 7 mm in diameter.

MTF–MEF was obtained by LAG of a mixture of MTF (0.5 mmol, 64.58 mg) and MEF (0.5 mmol, 120.64 mg) in a 1:1 stoichiometric ratio with 100 µL of ultrapure water as a liquid additive.

MTF–FLP and MTF–TLF were obtained using LAG of a mixture of MTF (0.5 mmol, 64.58 mg) and the respective coformer (130.85 mg of TLF, 122.13 mg of FLP) in a 1:1 stoichiometric ratio, along with 100 µL of ethanol as a liquid additive.

MTF–DIF and MTF–NIF hydrates were obtained using LAG of a mixture of MTF (0.5 mmol, 64.58 mg) and the respective coformer (125.10 mg of DIF, 141.11 mg of NIF) in a 1:1 stoichiometric ratio, along with 100 µL of ultrapure water as a liquid additive.

To obtain MTF–DIF and MTF–NIF as anhydrated salts, the product of the mechanochemical synthesis was heated at 100 °C for 2 h.

All reaction syntheses lasted for 30 min and were repeated to ensure reproducibility. Bulk materials were further evaluated using PXRD to determine the salt formation.

#### 2.2.3. Preparation of Single Crystals

Single crystals of MTF–based salts were obtained by dissolving the product of the LAGs in ethanol (for MTF–NIF·2H_2_O, MTF–MEF, MTF–FLP, and MTF–TLF) and ethyl acetate (for MTF–NIF). MTF–DIF single crystals were obtained using a hydrothermal reaction by placing a mixture of 0.2 mmol of MTF (25.8 mg) and 0.2 mmol of DIF (50.4 mg) in a hydrothermal reactor along with 3 mL of distilled water. The reactor was sealed and heated at 110 °C for 24 h. After cooling down to room temperature, the reactor was opened, and single crystals were separated from the solution for further characterization.

### 2.3. X-ray Diffraction Analysis

Single-crystal X-ray diffraction (SCXRD) data were acquired at room temperature on a Bruker D8 Venture diffractometer (Bruker-AXS, Karlsruhe, Germany) using CuKα radiation (λ = 1.54178 Å). The data were processed with the APEX4 suite [26]. The structures were solved with intrinsic phasing (SHELXT) [27] and refined with full-matrix least squares on F^2^ [28] using Olex2 as a graphical interface [29]. The non-hydrogen atoms were refined anisotropically. For all structures, hydrogen atoms were located in difference Fourier maps and included as fixed contributions riding on attached atoms with isotropic thermal displacement parameters 1.2 or 1.5 times those of the respective atom. Mercury [30], Platon [31], and Olex2 [29] were used for the analysis and visualization of the structures and also for graphic material preparation. All deposited CIF files are in the Cambridge Structural Database (CSD) under the CCDC numbers 2232928-2232933. Copies of the data can be obtained free of charge at https://www.ccdc.cam.ac.uk/structures/ (accessed on 29 December 2022).

Powder X-ray diffraction (PXRD) analysis was performed at room temperature on a Bruker D8 Advance Vαrio diffractometer (Bruker-AXS, Karlsruhe, Germany) diffractometer equipped with a LYNXEYE detector and CuKα_1_ radiation (1.5406 Å). The diffractograms were collected over an angular range of 5–40° (2θ) with a step size of 0.02° (2θ) and a constant counting time of 5 s per step.

### 2.4. Differential Scanning Calorimetry

A differential scanning calorimetry (DSC) study was performed with a Mettler-Toledo SC-822e calorimeter (Mettler Toledo, Columbus, OH, USA). Experimental conditions: aluminum crucibles of 40 µL volume, an atmosphere of dry nitrogen with 50 mL/min flow rate, and heating rates of 1 °C/min and 10 °C/min. The calorimeter was calibrated with indium of 99.99% purity (m.p.: 156.4 °C; DH: 28.14 J/g).

### 2.5. Stability Studies

Stability in an aqueous solution was evaluated through slurry experiments. An excess of powder samples of each phase was added to 1 mL of buffer phosphate (pH 6.8) and stirred for 24 h in sealed vials. The solids were collected, filtered, dried, and analyzed with PXRD.

Stability at accelerated aging conditions was also studied: 200 mg of solid was placed in watch glasses and left at 40 °C in 75% relative humidity using a Memmert HPP110 climate chamber (Memmert, Schwabach, Germany). Under the above-accelerated aging conditions, the stability of the solid forms was periodically monitored using PXRD for two months.

### 2.6. Solubility Studies

Samples for the solubility studies were prepared following the shake-flask method [32]. Saturated solutions were obtained by stirring an excess amount of APIs in 10 mL of pH 6.8 phosphate buffer at 25 °C until the thermodynamic equilibrium was reached after 24 h. Thermodynamic equilibrium was tested using UV spectroscopy by following the global solubility of the molecular salt over time until the plateau region was reached (3 h) and sustained more than 24 h. The solutions were then centrifuged, filtered through 0.22 μm polyether sulfone (PES) filters, and directly measured using high-performance liquid chromatography (HPLC). Appropriate dilutions were made to obtain measurable absorbance values. The absorbance measurements were thereafter used to quantify the MTF dissolved in each sample. The remaining solids were analyzed using PXRD to identify the crystalline phases and, thus, to check the stability of the initial crystalline phase.

HPLC experiments were performed with an Agilent 1200 HPLC system (Agilent Technologies, Santa Clara, CA, USA) equipped with a solvent degasser, pump, auto-sampler, and diode array detector. A Scharlau (Barcelona, Spain) 100 C18 chromatographic column (3 μm, 150 × 4.6 mm) was the thermostat at 25 °C and used for compound separation, using an isocratic elution method. The mobile phase was composed of a mixture of 10% acetonitrile (0.1% Formic acid, *v*/*v*) and 90% water (0.1% Formic acid, *v*/*v*). The flow rate was 1 mL/min, and the injection volume was 10 μL. The absorbance was measured at 233 nm, i.e., the maximum absorbance for metformin. Data acquisition and analysis were performed using the software ChemStation (Agilent Technologies, Santa Clara, CA, USA). The retention time for MTF was 1 min 54 s, and the concentration for the calibration curve was determined from the area under the MTF peak. The conditions are summarized in Supplementary Table S1.

## 3. Results and Discussion

### 3.1. Salt Synthesis

Mechanochemistry, especially liquid-assisted grinding (LAG), has been widely used in the pharmaceutical industry for the synthesis of new multicomponent materials. This methodology is well known to be efficient, quick, and reproducible, requiring a minimum amount of organic solvents compared with other traditional techniques [33].

**Figure 1:**
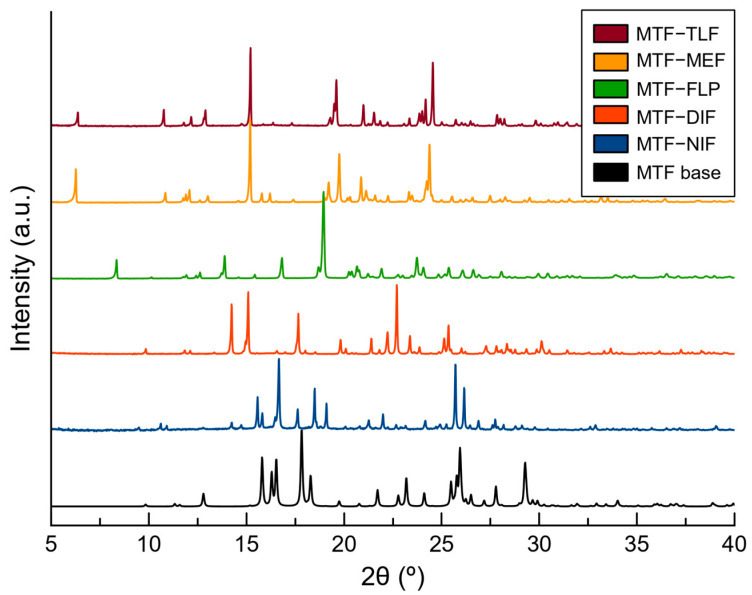
PXRD patterns of MTF base and the novel salts obtained by mechanochemical synthesis.

In this work, LAG reactions were performed using a 1:1 stoichiometric mixture of metformin base, previously neutralized, and the corresponding NSAIDs, along with ultrapure water or ethanol as an additive solvent. After 30 min of reaction, the powder obtained in each reaction was characterized using PXRD and compared with the corresponding parent APIs to evaluate the formation of the new salts. PXRD patterns demonstrated the formation of five new phases after LAG reactions and their purity (Figure 1 and Figure S1).

To obtain more information about the crystalline structure of the new phases, the polycrystalline products of the LAGs were used for recrystallization by dissolving them in ethanol and ethyl acetate. After slow solvent evaporation at room temperature, suitable crystals for single-crystal X-ray diffraction (SCXRD) were obtained.

From these experiments, MTF–TLF, MTF–MEF, MTF–FLP, and MTF–NIF structures were obtained. The structural information allowed the simulation of a calculated PXRD for each salt (Figure S1), which confirmed the phase purity of the bulk products obtained from LAG except for MTF–NIF, whose calculated powder pattern did not match the product obtained from the LAG reaction, thus indicating the formation of an intermediate phase or a hydrate phase. For that reason, the product of the LAG of MTF and NIF was heated at 100 °C for 2 h. After the thermal treatment, the PXRD pattern was in good agreement with the calculated powder pattern for MTF–NIF anhydrate salt. To confirm the formation of a hydrated phase during the LAG recrystallization, single crystals of MTF–NIF·2H_2_O were obtained and analyzed, which allowed the use of the calculated PXRD pattern to compare with the initial mechanochemical reaction. As expected, however, the PXRD patterns of MTF–NIF·2H_2_O and the product obtained from LAG synthesis did not match since they are different hydrated forms (Figure S2).

Similar results were obtained for MTF–DIF. Although good-quality single crystals were obtained from hydrothermal reactions, the bulk material obtained from LAG did not match the PXRD pattern of the anhydride phase. Following the previous methodology, the product of the LAG was heated at 100 °C for 2 h. After the treatment, the PXRD patterns matched perfectly. For MTF–DIF, no crystalline structure of a hydrate form was found.

### 3.2. Salt Screening

The Cambridge Structural Database (CSD version 5.43, update 4 from November 2022) was searched for MTF complexes resulting in 59 hits. After excluding entries corresponding to the MTF base molecule and its inorganic salts (4) as well as MTF metal complexes (29), the remaining dataset contained 26 molecular salts (44%). From the remaining molecular salts, 13 entries corresponded to MTF–drug salts, mainly antidiabetic drugs [34,35,36,37,38]. Only three salt structures containing MTF and an NSAID as counterion have been reported: MTF–diclofenac [39], MTF–salicylate [40], and MTF–aspirin [41] salts. The high number of observed molecular salts agrees with the strong basic nature of MTF. Table 1 evidences a significant difference in p*K*_a_ values between the ionizable groups of MTF and the selected NSAIDs. Thereby, proton transfer is expected from the carboxylic group present in NSAIDs to an amine moiety in MTF, according to the well-known p*K*_a_ rule widely used in the pharmaceutical industry [42,43,44]. Indeed, crystal structures of the multi-component drug–drug materials reported in this work confirm the molecular salt nature of these solids, which is consistent with other multicomponent pharmaceutical solids involving MTF published very recently [41,45,46].

### 3.3. Crystal Structure Analysis of Molecular Salts

Single crystals of molecular salts suitable for structural purposes were obtained, and their structures were determined using SCXRD. Crystallographic data for these salts are summarized in Table 2. Asymmetric units of the salts are represented in Figure S3, and hydrogen bond information is presented in Table S2. SCXRD data confirmed the proton transfer from the acid groups of NSAIDs to the basic nitrogen site of metformin. This finding was also evidenced in the experimental electron density map and confirmed by the analysis of the C–O bond distances of the carboxylate group of NSAIDs, with ΔD_C–O_ values being similar to those observed in salts, in the range 0.008–0.024 Å [53]. Due to the protonation of MTF, the resulting MTF^+^ cation is able to participate in hetero-synthons with the NSAID anion through guanidinium···carboxylate synthons, engaged through the $R_{2}^{2}\left(8\right)$ ring motif [54,55].

#### 3.3.1. MTF–MEF and MTF–TLF Salts

Molecular salts of MTF and the fenamic acids reported in this study (MEF and TLF) are isostructural (have the same crystal structure) and, in addition, isomorphous (have the same unit-cell dimensions and spacegroup [56]). The unit-cell parameters of two crystal structures were used to calculate the unit-cell similarity index Π (Equation (1)) [57]. A Π value of 0.003 confirms the isomorphous nature of the MTF–fenamate pairs. Moreover, PXRD similarity index scores for each pair and the RMSDs (root-mean-square deviations) were calculated from the packing similarity tool in Mercury [30] (overlay with 20 molecules and allowing molecular differences). The results obtained from these calculations showed that 20 out of 20 molecules were matched in the pairs of fenamate salts (PXRD similarity: 0.986; RMSD (Å): 0.131), suggesting that these molecular salts have identical intermolecular interactions and therefore affording the same crystal packing.

(1) $$\Pi =\frac{\left(a+b+c\right)}{\left(a^{\prime }+b^{\prime }+c^{\prime }\right)}-1$$

**Figure 2:**
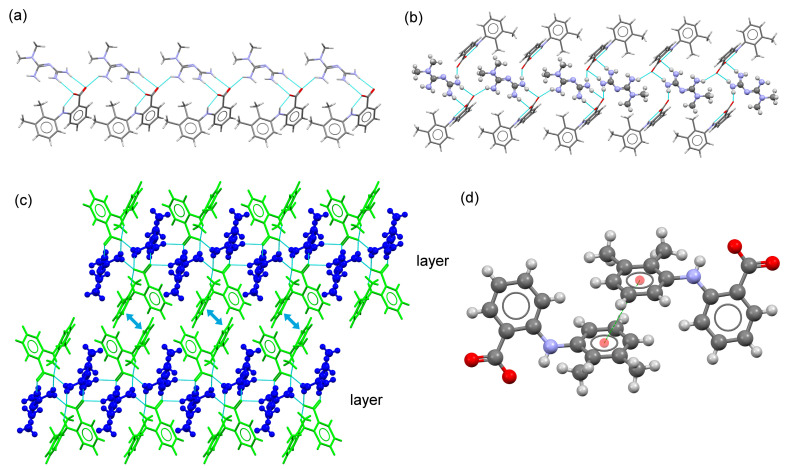
(**a**) Fragment of 1D-chain structure in the crystal structure of MTF–MEF. (**b**) Detailed view of the ribbon structure in the molecular salt MTF–MEF. MTF^+^ cations are represented as balls and sticks. (**c**) Detailed view of the packing arrangement of MTF cations (blue) and MEF anions (green) in the crystal structure of MTF–MEF. MTF^+^ cations are represented as balls and sticks. (**d**) A pair of stacked MEF–MEF anions.

These molecular salts crystallize in the monoclinic P2_1_/c space group, with one monoprotonated MTF^+^ cation and one fenamate (MEF or TLF) anion in the asymmetric unit as ionic pair. In the molecular salt, the ions are associated through the $R_{2}^{2}\left(8\right)$ ring motif built by the COO^−^ group and the terminal amines moiety of the MTF^+^ cation (Figure 2). Ionic pairs are aligned in a 1D-chain along [010] direction by the N–H⋯O H-bond (N2–H2D⋯O1) formed between the NH_2_ group in MTF^+^ cations and the COO^−^ group of fenamate anions. Along [001] direction, adjacent chains are held together through the $R_{4}^{2}\left(8\right)$ homo dimeric motif between two MTF^+^ fenamate pairs related by an inversion center, generating a ribbon structure. A layered 2D structure is further generated with π,π-stacking interactions between the substituted rings of fenamate ions. The supramolecular structure is then obtained by stacking these layers with H-bonds involving amine groups of MTF^+^ and carboxylate groups of fenamates (Figure 2c).

#### 3.3.2. MTF–NIF Salts and MTF–NIF·2H_2_O Hydrate Salt

**Figure 3:**
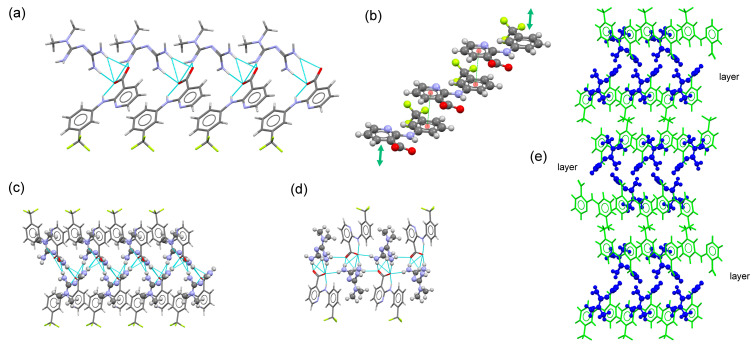
(**a**) Fragment of 1D-chain structure in the crystal structure of MTF–NIF. (**b**) Detailed view of multi-stacking π,π-interaction between NIF anions. (**c**) Detailed view of the ribbon structure in the molecular salt MTF–NIF. MTF^+^ cations are represented as balls and sticks. (**d**) Fragment of the 2D-layered structure in the molecular salt MTF–NIF. (**e**) Packing arrangement of MTF cations (blue) and NIF anions (green) in the crystal structure of MTF–NIF.

MTF–NIF salt crystallizes in the monoclinic system spacegroup P2_1_/n. The asymmetric unit of this crystal phase contains one MTF^+^ cation and a nifluminate anion. The ion pair is associated with a charge-assisted hydrogen bond involving the guanidium moiety of MTF and the carboxylate group of NIF. As in the previously described salt structures containing MEF and TLF, adjacent ion pairs are H-bonded to build a 1D chain (along the a-axis). π,π-stacking interactions between each one of the rings of NIF reinforce the chains. A 2D layered structure is then generated through H-bonds between amine groups of MTF cations and carboxylate groups of NIF anions. Finally, the supramolecular structure is obtained by stacking these layers (along the b-axis) facing –CF_3_ groups of NIF (Figure 3).

**Figure 4:**
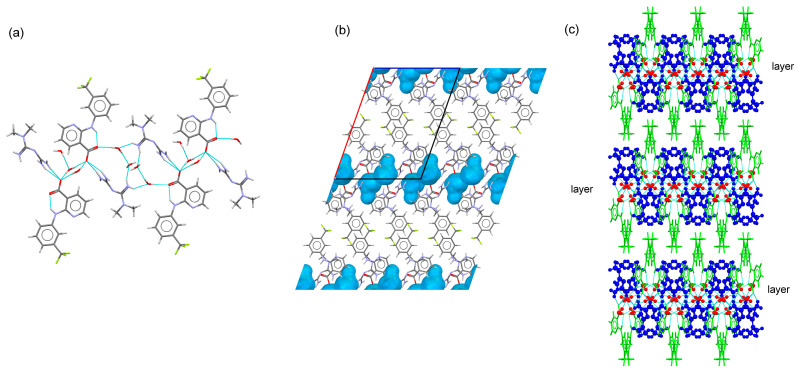
(**a**) Fragment of ribbon structure in the crystal structure of MTF–NIF·2H_2_O. (**b**) Detailed view voids where water molecules are located in MTF–NIF·2H_2_O. (**c**) Packing arrangement of MTF cations (blue), water molecules (red), and NIF anions (green) in the crystal structure of MTF–NIF·2H_2_O. MTF cations are represented as balls and sticks.

MTF–NIF·2H_2_O crystallized in the monoclinic system spacegroup P2_1_/c. The asymmetric unit of this crystal phase contains one MTF^+^ cation, a niflumic anion, and two water molecules, resulting in a salt hydrate. In the asymmetric unit, the ions are connected by charge-assisted hydrogen bonds between the guanidinium moiety of MTF and a carboxylate group of NIF. Unlike the previously described structures, this interaction involves only one oxygen atom from the carboxylate group of NIF ($R_{2}^{1}\left(6\right)$ graph set). Water molecules and ion pars are connected by H-bonding interactions to build a ribbon structure that extends along the b-axis, locating the –CF_3_ groups in the periphery. Additional H-bonding interactions involving the terminal amino group of the MTF^+^ cation and water molecules connect ribbons to build a 2D layered structure that extends parallel to the bc- plane of the crystal. Finally, the supramolecular 3D structure is generated by stacking these layers along the a-axis facing the -CF_3_ containing rings of NIF anions (Figure 4).

**Figure 5:**
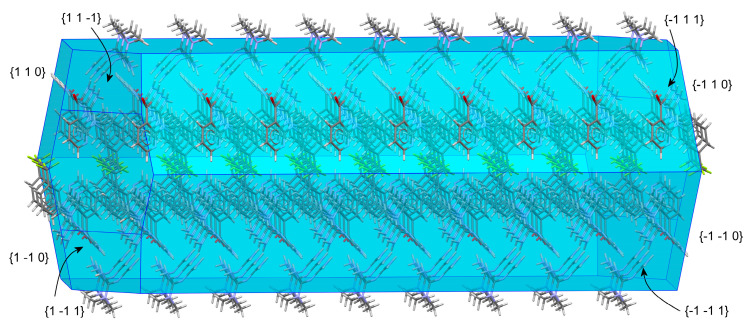
BFDH−predicted morphology of MTF–NIF salt.

A possible explanation for the transformation of the anhydrate salt into the hydrate salt form can be obtained from the study of the predicted crystal morphology of MTF–NIF (Figure 5). BFDH morphology of the anhydrate MTF–NIF salt was calculated by using the Bravais–Friedel–Donnay–Harker (BFDH) method included in the latest release of the visualization software package Mercury [30]. In the case of MTF–NIF, the faces {−110}, {−1–10}, {1 1 0} and {1 −1 0} (corresponding to 14.4 % of the total surface) and the faces {1 −1 −1}, {1 1 −1}, {−1 −1 1} and {−1 1 1} (corresponding to 32 % of the total surface) expose amino groups of MTF and carboxylate groups of NIF to the surface and coincide with the crystal structure region where the ion pairs form the tape-like structures. Therefore, it seems reasonable that water molecules can access and form additional H-bonds with the groups involved resulting in the hydrated structure as reported herein.

#### 3.3.3. MTF–DIF Salt

**Figure 6:**
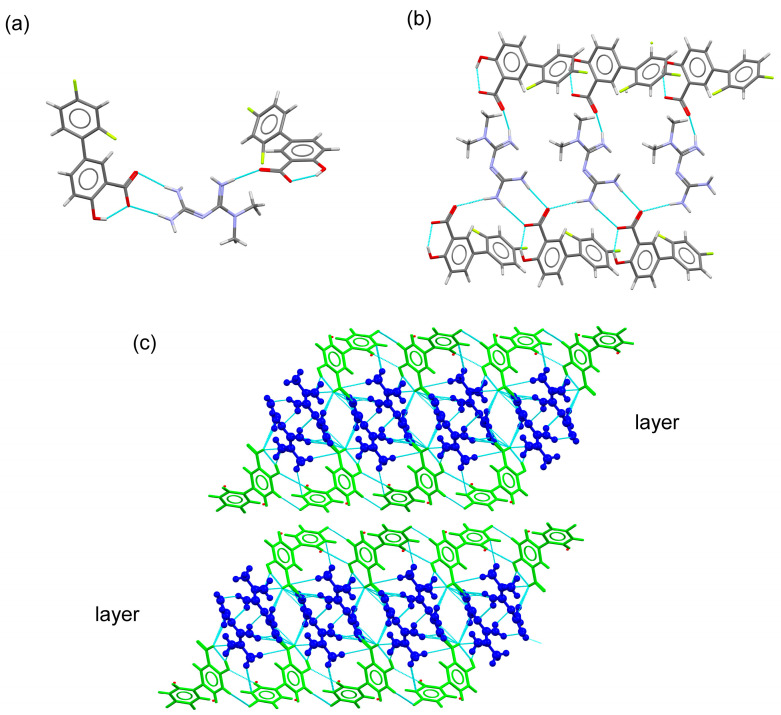
(**a**) Trimer structure generated by H-bonding interactions in the MTF–DIF salt. (**b**)Fragment of the ribbon structure generated using connecting trimers with H-bonding interactions. (**c**) Detailed view of the packing arrangement of MTF cations (blue) and DIF anions (green) in the crystal structure of MTF–DIF. MTF cations are represented as balls and sticks.

MTF–DIF crystallized in the monoclinic system spacegroup C2/c, with one MTF^+^ cation and one DIF anion in the asymmetric unit. A trimer was formed by MTF and two DIF ions through guanidinium···carboxylate, N4–H4E⋯O2 (3.01 Å) and N5–H5B⋯O1 (2.96 Å) ($R_{2}^{2}\left(8\right)$ graph set), and N2–H2A···O2 (2.92 Å) hydrogen bonds (Figure 6). The trimers are then linked to build a ribbon structure through N5–H5A⋯O2 (3.05 Å) hydrogen bonds along the b-axis (Figure 6b). Additional H-bond involving amine groups of MTF^+^ and carboxylate groups of DIF generate a 2D layered structure running parallel to the bc-plane of the crystal. C-H···F contacts participate in the cohesion of these structures. The supramolecular structure is finally built with stacks of layers facing 2,4-difluorophenyl rings of DIF (Figure 6).

#### 3.3.4. MTF–FLP Salt

**Figure 7:**
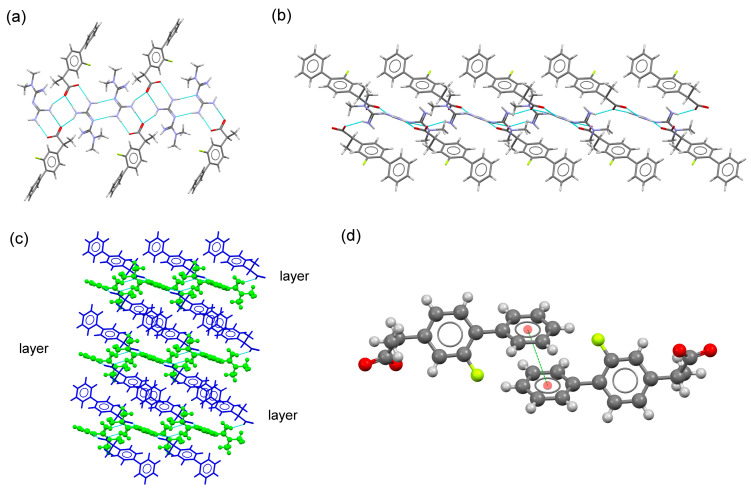
(**a**) Fragment of ribbon structure in the crystal structure of MTF–FLP. (**b**) Fragment of the 2D-layered structure in the molecular salt MTF–FLP. (**c**) Packing arrangement of MTF cations (blue) and NIF anions (green) in the crystal structure of MTF–DIF. (**d**) A pair of stacked FLP–FLP anions.

This molecular salt crystallizes in the triclinic system space group P-1, with one monoprotonated MTF^+^ cation and one FLP anion in the asymmetric unit as ionic pair. Both ions are associated with a charge-assisted H-bond through the guanidinium moiety of MTF^+^ and the carboxylate group of FLP anion ($R_{2}^{2}\left(8\right)$ graph set). Adjacent pairs are further connected by H-bonds ($R_{4}^{2}\left(8\right)$ graph set) to build a ribbon structure that extends along the c-axis, locating the FLP ions outside. 2D-layered structures are then generated by connecting ribbons involving non-terminal amine groups of MTF^+^ and carboxylate groups. The 3D supramolecular architecture is built through π,π-stacking interactions between the non-substituted aromatic ring of FLP anions that connect adjacent layers (Figure 7).

### 3.4. Thermal Analysis

**Figure 8:**
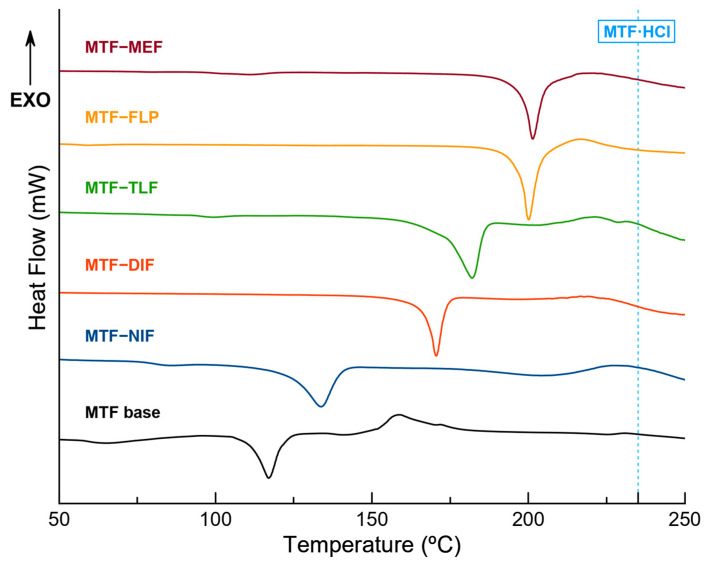
DSC traces of the pure molecular salts and MTF base. Blue-dotted line corresponds to the melting point of MTF·HCl.

Differential scanning calorimetry (DSC) was used to evaluate the thermal stability and determine the melting point of the new phases. Figure 8 shows the DSC traces and the endothermic events occurring during the experiments, which correspond to the melting point of the salts. The existence of one single and well-defined endothermic event confirms the purity of the phases, which is in good agreement with the results obtained using PXRD. In addition, the presence of only one endothermic event demonstrates the stability of the phase under the melting point. Over the transition state, other endothermic events are observed, ascribed to the degradation of the samples.

With the exception of MTF–FLP, all the molecular salts show a melting point in a range between the melting point of the MTF base (117 °C) and the corresponding NSAID coformer (MEF 230 °C, DIF 210–211 °C, TLF 207 °C, NIF 204 °C), increasing the thermal stability of the MTF base in all cases, although still under the melting point of the commercially available MTF·HCl (231.5 °C) [46]. This behavior has already been described by other researchers, demonstrating the ability of multicomponent materials to modulate the thermal behavior of APIs [58]. Interestingly, the melting point of MTF–FLP is 200 °C, while the melting point of the components goes from 110 to 117 °C, increasing the thermal stability and the melting point of both APIs by more than 80 °C.

### 3.5. Stability Studies

The thermodynamic stability of molecular salts was studied by conducting aqueous slurry experiments at 25 °C. After 24 h, the suspensions were filtered, air-dried at room temperature, and characterized using PXRD (Figure S4). No observable changes in color or texture were observed for the samples. Despite MTF–NIF and MTF–DIF displaying a phase transition indicating low stability in solution, MTF–TLF, MTF–MEF, and MTF–FLP remained stable upon slurrying, as the crystallinity and the initial crystal structures remained intact. SCXRD and further PXRD confirmed the formation of the dihydrate salt of MTF–NIF, but it was impossible to determine the final phase of MTF–DIF. Interestingly, this unknown phase agrees with the phase obtained directly from the LAG reaction using water as a liquid additive (Figure S5), suggesting the formation of a hydrate form of MTF–DIF salt.

The molecular salts were also stored under accelerated aging conditions (40 °C and 75% RH). Under these conditions, all the samples remained stable after four months, with the exception of MTF–NIF (Figure S6), which presented a partial phase transition on the third day, with a final conversion at one week. This new phase matched neither the already determined dihydrate salt form nor the product of the LAG in water, suggesting the formation of different grades of hydrate salt for the MTF–NIF system.

### 3.6. Solubility Studies

Solubility and oral bioavailability are closely related. Therefore, solubility enhancement is one of the most common approaches to increasing the bioavailability of poorly soluble drugs [59,60,61].

In this work, HPLC was used to determine the solubility of the proposed MTF molecular salts. The solubility improvement of NSAID coformers was also assessed through data normalization, using the corresponding stoichiometry and molecular weight of MTF-NSAID salts and isolated components. It should be noted that this procedure can only be applied to stable phases in which the stoichiometry MTF:NSAID is maintained.

Low stability was observed for MTF–NIF, and MTF–DIF in the stability test performed in aqueous media. A phase transformation for MTF–NIF was confirmed through the obtention of MTF–NIF·2H_2_O single crystals. For this reason, the solubility of MTF–NIF could not be determined at the reported experimental conditions. Instead, the solubility corresponding to the MTF–NIF salt hydrate was obtained. On the other hand, the phase transformation for MTF–DIF could not be determined, thus, making MTF–DIF unsuitable for solubility studies.

Table 3 shows a notorious increase in the solubility of NSAIDs when compared with the reported isolated APIs: MTF–TLF increased solubility 111 times, MTF–MEF 208 times, MTF–NIF·2H_2_O 574 times, and finally MTF–FLP 1110 times, while exhibiting good stability. Furthermore, an interesting modulation of MTF solubility is also observed in the novel multicomponent molecular salts when compared with the solubility of MTF·HCl (ranging from 250 mg/mL [46,62,63]). In all reported MTF–NSAID salts, the solubility is reduced more than 50 times, with MTF–TLF decreasing the solubility of MTF by 90 times. Bearing in mind that clinical side effects of metformin were mainly caused by the gastrointestinal accumulation of MTF·HCl due to its extreme solubility [12], the obtention of a less soluble phase might reduce such side effects.

The remaining solids were finally analyzed using PXRD to identify the crystalline phases, thus confirming the stability of the initial phases and the congruent solubility of these new salts.

The remarkable solubility values obtained for the novel MTF–NSAID salts are consistent with the formation of molecular salts [7], as already expected from their p*K*_a_ values. Furthermore, it is well known that the physicochemical properties of solids, such as solubility, are also strongly dependent on their intimate crystal structure. Herein, the layered arrangement exhibited by the APIs and the presence of charge-assisted hydrogen bonds in the crystal structure [64,65] are eventually responsible for the new solubility properties.

**Table 3:** HPLC solubilities of the pure salts compared with their corresponding components.

Compound	MTF Solubility in the Salts [mg/mL]	NSAID Solubility in the Salts [mg/mL]	Solubility Enhancement over MTF·HCl/NSAID	NSAID Solubility [mg/mL]	Ref.
MTF–NIF·2H_2_O	5.290	11.493	0.0212x/574x	<0.02	[66]
MTF–FLP	4.775	5.552	0.0191x/1110x	<0.005	[67]
MTF–MEF	3.185	5.950	0.0127x/119x	<0.05	[68,69]
MTF–TLF	2.740	10.397	0.0110x/208x	<0.05	[68]

## 4. Conclusions

The novel multicomponent MTF–NSAID solids succeeded in overcoming two of the main problems that these APIs have when administered separately. On the one hand, MTF stability has been improved while achieving an outstanding solubility profile. For instance, the reported MTF–NSAID solids are more soluble than MTF base but less soluble than MTF·HCl salt, thus potentially reducing improper intestinal accumulation, which is one of the most common side effects associated with the current MTF·HCl chronic treatment. On the other hand, MTF–NSAID molecular salts, thanks to the salification strategy, are able to significantly enhance the solubility of NSAIDs, thus reducing the dosage and the undesired gastrointestinal dose-dependent side effects of these drugs.

Interestingly, structure-properties relationships could be gathered from the thorough study of the intimate crystal structure of the new multicomponent MTF–NSAIDs molecular salts. The disruption of the robust NSAID–NSAID dimers present in the crystal structure of isolated NSAIDs is key for understanding their enhancement in solubility. Moreover, the new layered NSAID–MTF–NSAID structure in the novel multicomponent materials protects MTF from water, explaining the higher stability and the new solubility profile.

Considering all the above, the novel drug–drug molecular salts are worthy of further investigation. Our results confirm improved physicochemical properties thanks to the novel formulation, among which a better solubility profile should be remarked. Improving solubility should not be underestimated since a poor solubility profile is currently the most important limitation of oral biopharmaceutics in the pharmaceutical industry. Likewise, optimized solubility opens the door to dosage reduction and consequently might reduce those side effects associated with MTF–NSAID treatments.

## Figures and Tables

**Table 1:** Molecules used to make salts with MTF and their corresponding p*K*_a_ values.

APIs	Reported p*K*_a_ Values	References	Calculated p*K*_a_ Values *	Calculated MTF-NSAID Δp*K*_a_
Metformin	12.4	[47]	12.30	-
Mefenamic Acid	3.93	[48]	3.89	8.41
Tolfenamic Acid	4.3	[49]	3.88	8.42
Niflumic acid	1.88	[50]	1.88	10.42
Diflunisal	2.69	[50]	2.69	9.61
Flurbiprofen	4.2	[51]	4.42	7.88

* Estimated using the p*K*_a_ plugin in MarvinSketch software [52].

**Table 2:** Crystallographic data and structure refinement details of MTF—NSAIDs molecular salts.

Compound Name	MFT–MEF	MFT–TLF	MFT–NIF	MFT–NIF·2H_2_O	MTF–DIF	MFT–FLP
Formula	C_19_H_26_N_6_O_2_	C_18_H_23_ClN_6_O_2_	C_17_H_20_F_3_N_7_O_2_	C_17_H_24_F_3_N_7_O_4_	C_17_H_19_F_2_N_5_O3	C_19_H_24_FN_5_O_2_
Formula weight	370.46	390.87	411.40	447.43	379.37	373.43
Crystal system	Monoclinic	Monoclinic	Monoclinic	Monoclinic	Monoclinic	Triclinic
Space group	P2_1_/c	P2_1_/c	P2_1_/n	P2_1_/c	C2/c	P-1
a/Å	15.5701(19)	15.5584(15)	6.2011(16)	19.9296(15)	36.998(2)	8.7587(5)
b/Å	8.7141(11)	8.7072(8)	32.216(8)	7.9245(6)	6.7220(5)	10.5391(7)
c/Å	16.239(2)	16.3668(16)	9.846(3)	14.6836(11)	15.0427(9)	10.5990(6)
α/°	90	90	90	90	90	90.265(2)
β/°	116.408(4)	117.919(4)	101.051(13)	109.362(4)	104.981(3)	96.217(2)
γ/°	90	90	90	90	90	94.624(2)
V/Å^3^	1973.4(4)	1959.2(3)	1930.6(8)	2187.9(3)	3614.0(4)	969.37(10)
Z	4	4	4	4	8	2
D_c_/g cm^−3^	1.247	1.325	1.415	1.358	1.394	1.279
F(000)	792	824	856	936	1584	398
Reflections collected	20,802	19,113	19,888	17,437	18,152	14,188
Unique reflections	3509	3452	3396	3853	3155	3366
Data/ restraints/parameters	3509/0/251	3452/0/248	3396/0/264	3853/0/288	3155/5/258	3366/0/248
Goodness-of-fit on F^2^	1.053	1.048	1.036	1.026	1.037	1.068
*R*1 (I > 2σ(I))	0.0398	0.0477	0.0640	0.0633	0.0380	0.0551
w*R*2 (I > 2σ(I))	0.1153	0.1315	0.1758	0.1673	0.1044	0.1622

## Data Availability

The Crystallographic Information File with the structural data of the new phase can be obtained from the CCDC and requested with the references 2232928-2232933 at https://www.ccdc.cam.ac.uk/structures/ (accessed on 29 December 2022).

## Supplementary Materials

The following supporting information can be downloaded at: https://www.mdpi.com/article/10.3390/pharmaceutics15020449/s1, Figure S1: PXRD patterns of the molecular salts obtained using LAG, compared with their respective components; Figure S2: PXRD pattern of MTF–NIFH2O, compared with the product of the LAG in water; Figure S3: ORTEP representation showing the asymmetric unit of MTF—MEF (a), MTF—TLF (b), MTF—NIF (c), MTF—NIF·2H2O (d), MTF—DIF (e), and MTF—FLP (f) with an atom numbering scheme (thermal ellipsoids are plotted with a 50% probability level); Figure S4: PXRD patterns of the reported molecular salts after aqueous slurrying for 24 h; Figure S5: PXRD pattern of LAG of the mixture of MTF and DIF using water as liquid additive compared with the product after the slurry of MTF—DIF in aqueous media; Figure S6: PXRD patterns of reported molecular salts under accelerated aging conditions for 4 months; Figure S7: Calibration curve of MTF·HCl determined from HPLC data; Table S1: HPLC method parameters; Table S2: Hydrogen bonds for MTF—NSAIDs molecular salts [Å and deg.]; Table S3: π,π-stacking interactions analysis of compounds MTF—MEF, MTF—TLP, and MTF—FLP.
