# Supplementary material for: Metformin-NSAIDs Molecular Salts: A Path towards Enhanced Oral Bioavailability and Stability
Source: Pharmaceutics. 2023 Jan 29;15(2):449. doi: 10.3390/pharmaceutics15020449 (PMC9966766; doi:10.3390/pharmaceutics15020449)
Supplement: Supplementary file 1 [file pharmaceutics-15-00449-s001.zip › pharmaceutics-2161347-supplementary.pdf]

Article

Metformin-NSAIDs molecular salts: a path towards enhanced oral bioavailability and stability

Francisco Javier Acebedo-Martínez, Alicia Domínguez-Martín, Carolina Alarcón-Payer, Carolina Garcés-Bastida, Cristóbal Verdugo-Escamilla, Jaime Gómez-Morales, Duane Choquesillo-Lazarte*

Table of contents

Figure S1. PXRD patterns of the molecular salts obtained by LAG, compared with their respective components.
Figure S2. PXRD pattern of MTF–NIFH_2O, compared with the product of the LAG in water.
Figure S3. ORTEP representation showing the asymmetric unit of MTF—MEF (a), MTF—TLF (b), MTF—NIF (c), MTF—NIF·$2H_2O$ (d), MTF—DIF (e), and MTF—FLP (f) with atom numbering scheme (thermal ellipsoids are plotted with the 50% probability level).
Figure S4. PXRD patterns of the reported molecular salts after aqueous slurrying for 24 hours.
Figure S5. PXRD pattern of LAG of the mixture of MTF and DIF using water as liquid additive compared with the product after the slurry of MTF—DIF in aqueous media.
Figure S6. PXRD patterns of reported molecular salts under accelerated ageing conditions for 4 months.
Figure S7. Calibration curve of MTF·HCl determined from HPLC data.

Table S1. HPLC method parameters.
Table S2. Hydrogen bonds for MTF—NSAIDs molecular salts [Å and deg.].
Table S3. π,π-stacking interactions analysis of compounds MTF—MEF, MTF—TLP and MTF—FLP.

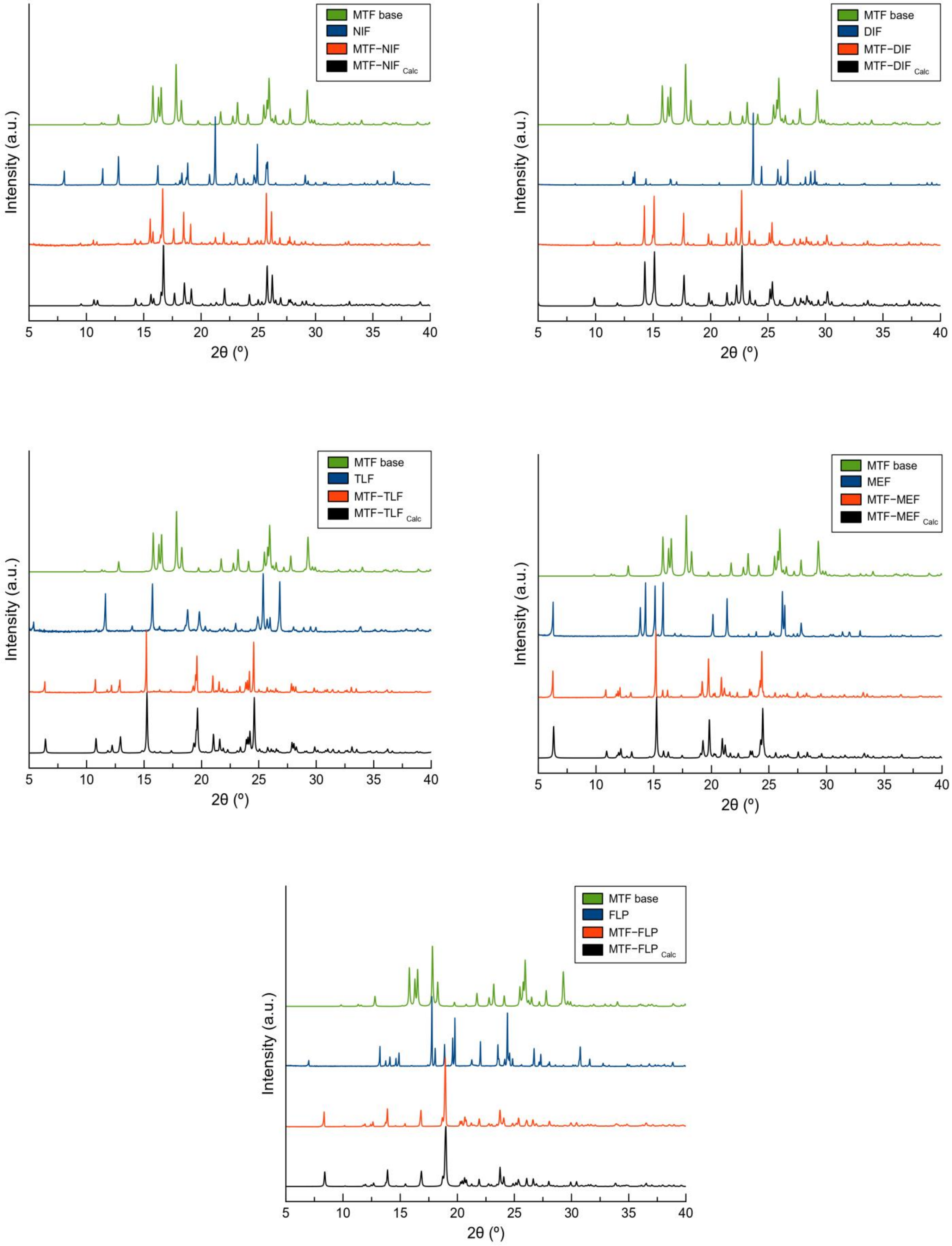

Figure S1. PXRD patterns of the molecular salts obtained by LAG, compared with their respective components.

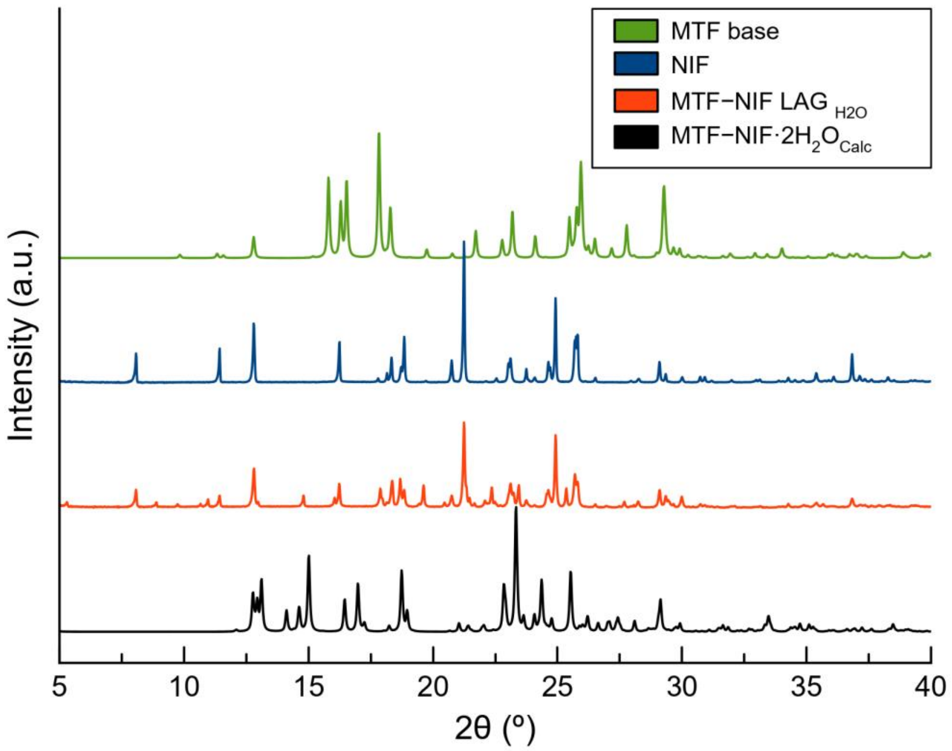

Figure S2. PXRD patterns of MTF–NIF·$2H_2O$, compared with the product of the LAG in water.

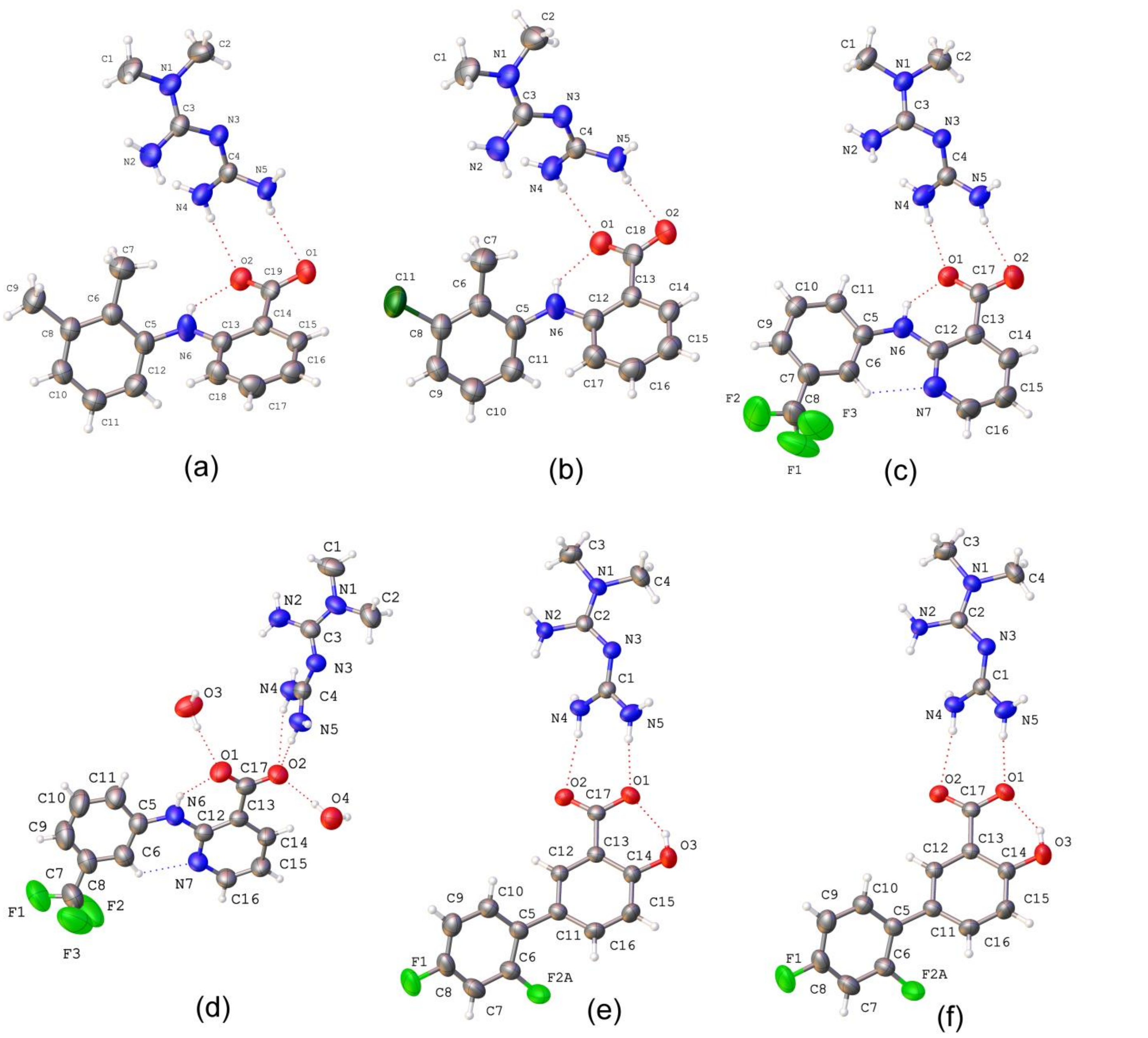

Figure S3. ORTEP representation showing the asymmetric unit of MTF—MEF (a), MTF—TLF (b), MTF—NIF (c), MTF—NIF·$2H_2O$ (d), MTF—DIF (e), and MTF—FLP (f) with atom numbering scheme (thermal ellipsoids are plotted with the 50% probability level).

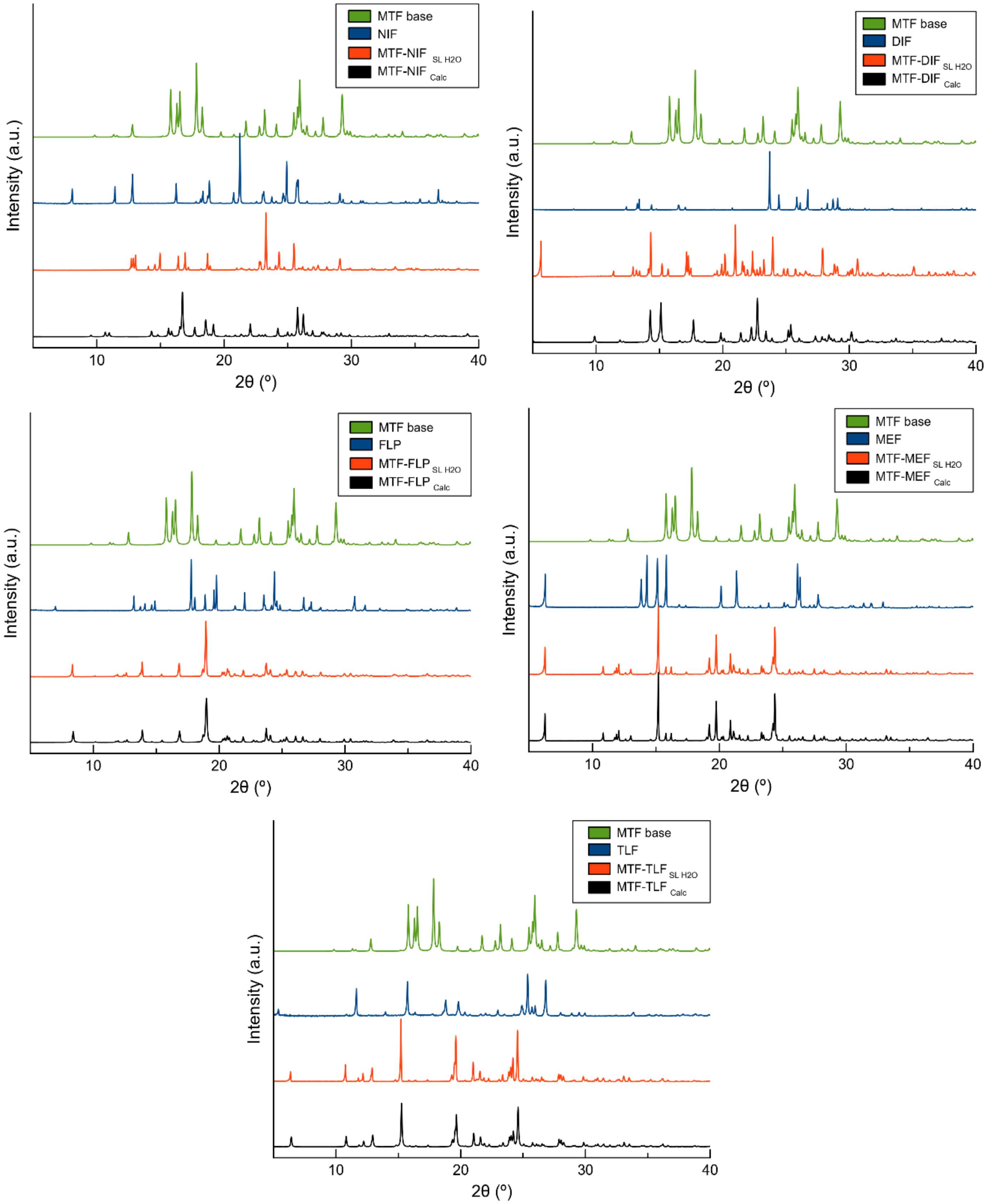

Figure S4. PXRD patterns of the reported molecular salts after aqueous slurrying for 24 hours.

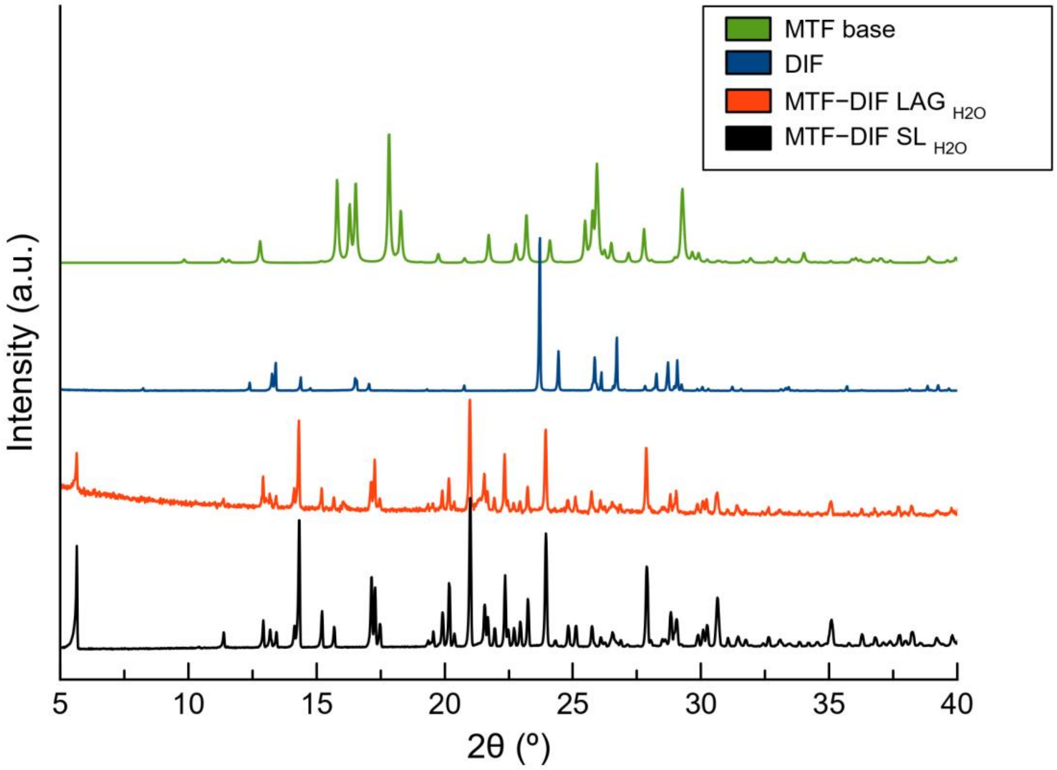

Figure S5. PXRD pattern of LAG of the mixture of MTF and DIF using water as liquid additive compared with the product after the slurry of MTF—DIF in aqueous media.

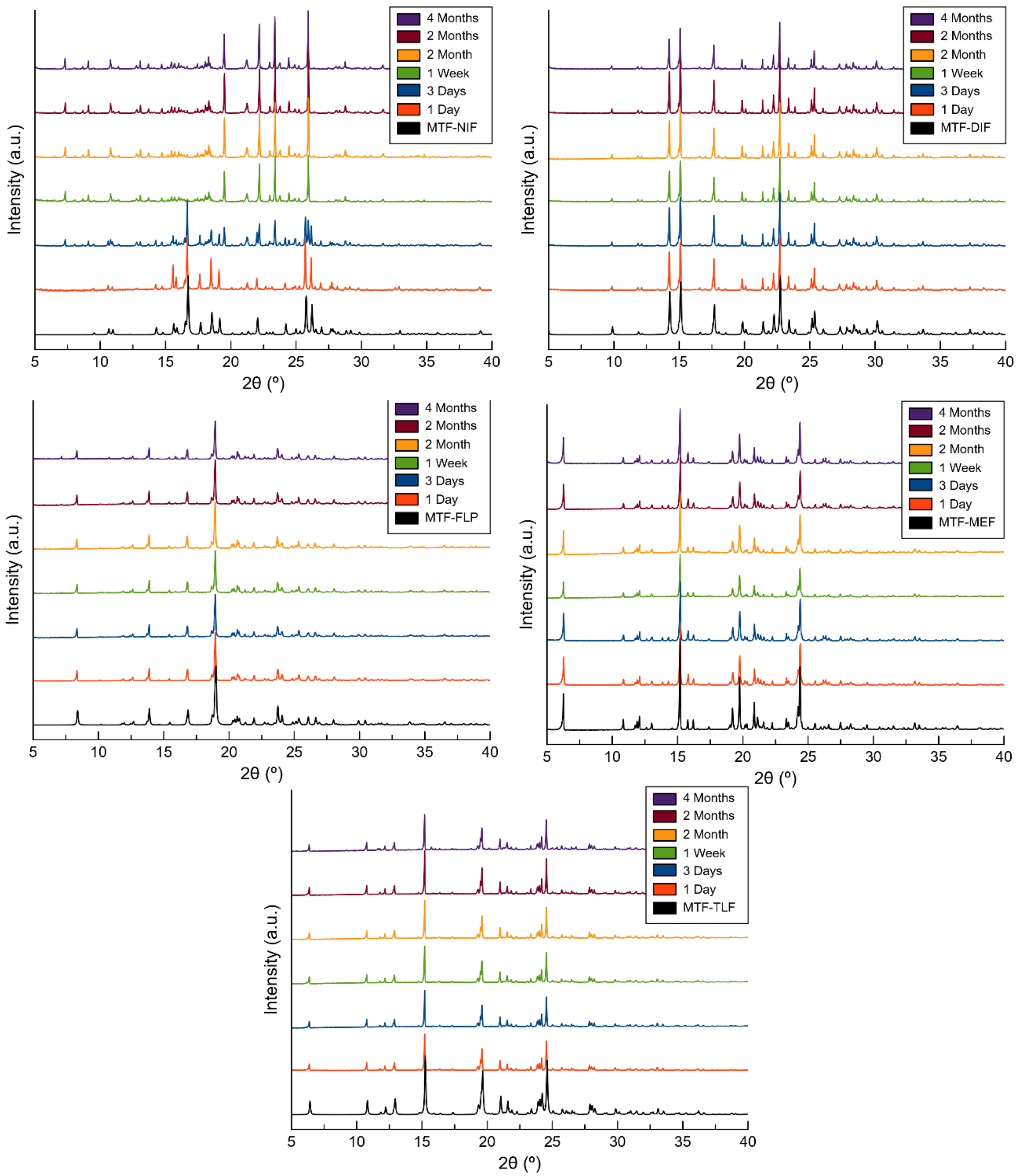

Figure S6. PXRD patterns of reported molecular salts under accelerated ageing conditions for 4 months.

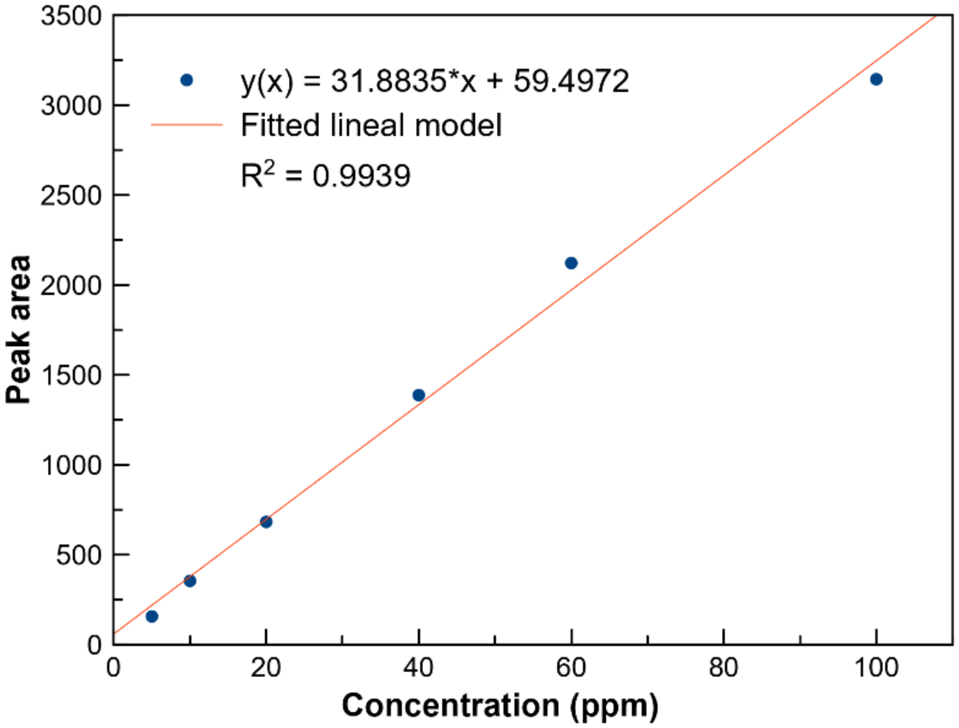

Figure S7. Calibration curve of MTF·HCl determined from HPLC data.

Table S1. HPLC method parameters

Parameter	Details
Column	100 C18 (4.6 mm × 150 mm, 3 μm particle size) Scharlau
Mobile Phase	Isocratic 10% phase A : 90% phase B.
	A= 10% acetonitrile (0.1% Formic acid, v/v) B= 90% water (0.1% Formic acid, v/v).
Flow Rate	1 mL/min
Injected Volume	10 μL
Column Temperature	25 ºC
Sample Temperature	25 ºC
λ_{max}	233 nm
Retention Time	1 minute 54 seconds
Equation	y(x) = 31.8835*x + 59.4972
Regression Coefficient (R^2)	0.9939
Calibration Range	5– 100 mg/L (ppm)

Table S2. Hydrogen bonds for MTF—NSAIDs molecular salts [Å and deg.].

	D-H···A	d(D-H)	d(H···A)	d(D···A)	<(DHA)
**MTF-MEF**	N(2)-H(2D)···O(1)	0.86	2.17	2.8455(16)	135.4
	N(4)-H(4A)···O(2)#1	0.86	2.15	2.9671(16)	157.9
	N(4)-H(4B)···O(2)#2	0.86	2.11	2.9165(16)	156.1
	N(5)-H(5A)···O(1)#3	0.86	2.11	2.9049(18)	151.8
	N(5)-H(5B)···O(1)#1	0.86	2.23	2.9794(17)	144.9
	N(6)-H(6)···O(2)	0.86	1.96	2.6496(17)	135.7
	#1 x,y+1,z #2 -x+1,y+1/2,-z+3/2 #3 -x+1,-y+2,-z+1				
**MTF-TLF**	D-H···A	d(D-H)	d(H···A)	d(D···A)	<(DHA)
	N(2)-H(2E)···O(2)	0.86	2.09	2.839(2)	144.9
	N(4)-H(4A)···O(1)#1	0.86	2.16	2.969(2)	157.3
	N(4)-H(4B)···O(1)#2	0.86	2.12	2.930(2)	156.9
	N(5)-H(5A)···O(2)#3	0.86	2.12	2.911(3)	152.0
	N(5)-H(5B)···O(2)#1	0.86	2.16	2.988(2)	161.5
	N(6)-H(6)···O(1)	0.86	1.95	2.637(2)	136.5
	#1 x,y-1,z #2 -x+1,y-1/2,-z+3/2 #3 -x+1,-y,-z+1				
**MTF-NIF**	D-H···A	d(D-H)	d(H···A)	d(D···A)	<(DHA)
	C(6)-H(6)···N(7)	0.93	2.29	2.883(5)	121.5
	N(2)-H(2D)···N(4)#1	0.86	2.77	3.399(4)	131.1
	N(2)-H(2E)···O(1)#1	0.86	2.60	3.236(4)	131.2
	N(2)-H(2E)···O(2)#1	0.86	2.18	3.035(4)	174.1
	N(4)-H(4A)···O(2)#2	0.86	2.27	3.063(3)	153.3
	N(4)-H(4B)···O(1)	0.86	1.98	2.828(3)	167.6
	N(5)-H(5A)···O(1)#3	0.86	2.14	2.950(3)	156.3
	#1 x-1,y,z #2 x-1/2,-y+1/2,z+1/2 #3 x-1/2,-y+1/2,z-1/2				

Table S2 (cont.). Hydrogen bonds for MTF—NSAIDs molecular salts [Å and deg.].

**MTF-NIF·H_2O**	D-H···A	d(D-H)	d(H···A)	d(D···A)	<(DHA)
	N(2)-H(2D)···O(3)#1	0.86	2.18	2.923(3)	145.0
	N(2)-H(2E)···O(4)#2	0.86	2.31	3.003(3)	138.3
	N(4)-H(4A)···O(1)	0.86	2.15	2.915(3)	147.2
	N(4)-H(4B)···O(2)#3	0.86	2.43	3.195(3)	148.1
	N(5)-H(5A)···O(3)#4	0.86	2.23	3.032(4)	156.1
	N(5)-H(5B)···O(2)#3	0.86	2.25	3.057(3)	155.6
	C(6)-H(6)···N(7)	0.93	2.30	2.893(4)	120.8
	N(6)-H(6A)···O(1)	0.86	1.91	2.628(3)	139.6
	O(3)-H(3A)···O(1)	0.85	1.90	2.742(3)	170.9
	O(3)-H(3B)···O(4)#5	0.85	1.97	2.816(3)	170.3
	O(4)-H(4C)···O(2)	0.85	1.96	2.808(3)	171.3
	O(4)-H(4D)···O(2)#6	0.85	2.00	2.838(3)	166.4
	#1 x,-y+1/2,z+1/2 #2 -x+2,-y+1,-z+1 #3 -x+2,y+1/2,-z+1/2				
	#4 x,y+1,z #5 x,-y+1/2,z-1/2 #6 -x+2,-y,-z+1				
**MTF-DIF**	D-H···A	d(D-H)	d(H···A)	d(D···A)	<(DHA)
	N(2)-H(2A)···O(2)	0.86	2.11	2.9209(15)	156.9
	N(2)-H(2B)···N(3)#1	0.86	2.43	3.1618(17)	143.5
	N(4)-H(4E)···O(2)#1	0.86	2.16	3.0116(15)	152.7
	N(5)-H(5A)···O(2)#2	0.86	2.20	3.0505(18)	169.2
	N(5)-H(5B)···O(1)#1	0.86	2.17	2.9569(17)	151.3
	C(15)-H(15)···F(1)#3	0.93	2.57	3.180(2)	123.7
	O(3)-H(3)···O(1)	0.82	1.81	2.5367(15)	146.7
	#1 -x+1/2,y+1/2,-z+1/2 #2 -x+1/2,y-1/2,-z+1/2 #3 x,-y+2,z+1/2				
**MTF-FLP**	D-H···A	d(D-H)	d(H···A)	d(D···A)	<(DHA)
	N(2)-H(2A)···O(1)#1	0.86	2.11	2.866(2)	146.6
	N(4)-H(4A)···O(2)	0.86	1.95	2.791(3)	165.6
	N(4)-H(4B)···O(2)#2	0.86	2.07	2.861(3)	152.6
	N(5)-H(5A)···O(1)	0.86	2.01	2.867(3)	175.5
	N(5)-H(5B)···N(3)#3	0.86	2.30	3.152(3)	171.3
	#1 x+1,y,z #2 -x+2,-y+1,-z+1 #3 -x+2,-y+1,-z+2				

Table S3. π,π-stacking interactions analysis of compounds MTF—MEF, MTF—TLP and MTF—FLP.

MTF—MEF

6-Membered Ring (1)   C5 --> C6 --> C8 --> C10 --> C11 --> C12 -->

6-Membered Ring (2)   C13 --> C14 --> C15 --> C16 --> C17 --> C18 -->

Cg(I)	Res(I)		Cg(J)	[ARU(J)]	Cg-Cg	Transformed J-Plane P	Q	R	S	Alpha	Beta	Gamma	CgI_Perp	CgJ_Perp	Slippage	
Cg1	[1]	->	Cg1	[3556.01]	4.0614(11)	-0.0357	-0.1565	-0.9870	-5.2777	0.02(8)	29.3	29.3	3.5431(7)	3.5431(7)	1.985	**MEF-MEF**
Cg1	[1]	->	Cg2	[4555.01]	5.0184(11)	0.6398	0.6413	-0.4236	-8.4282	72.85(8)	25.9	46.9	3.4282(7)	4.5130(6)		
Cg2	[1]	->	Cg1	[3566.01]	5.4305(13)	-0.0357	-0.1565	-0.9870	-6.6412	60.29(8)	33.4	86.3	0.3495(6)	4.5334(7)		
Cg2	[1]	->	Cg1	[4554.01]	5.0184(11)	0.0357	-0.1565	0.9870	1.0898	72.85(8)	46.9	25.9	4.5129(6)	3.4282(7)		
				Min or Max	4.061					0.0	25.9	86.3	-3.543	-4.533		

[3556] = -X,-Y,1-Z
[4555] = X,1/2-Y,1/2+Z
[3566] = -X,1-Y,1-Z
[4554] = X,1/2-Y,-1/2+Z

MTF—TLF

6-Membered Ring (1)   C5 --> C6 --> C8 --> C9 --> C10 --> C11 -->

6-Membered Ring (2)   C12 --> C13 --> C14 --> C15 --> C16 --> C17 -->

Cg(I)	Res(I)		Cg(J)	[ARU(J)]	Cg-Cg	Transformed J-Plane P	Q	R	S	Alpha	Beta	Gamma	CgI_Perp	CgJ_Perp	Slippage	
Cg1	[1]	->	Cg1	[3576.01]	3.9352(16)	-0.0211	0.1851	-0.9825	-3.6984	0.00(12)	29.4	29.4	3.4277(10)	3.4276(10)	1.933	**TLF-TLF**
Cg1	[1]	->	Cg2	[4565.01]	5.0454(15)	0.6334	-0.6491	-0.4212	-14.2733	73.72(12)	28.6	45.1	3.5585(12)	4.4308(10)		
Cg2	[1]	->	Cg1	[3566.01]	5.3150(17)	-0.0211	0.1851	-0.9825	-5.3098	58.63(12)	32.1	89.2	0.0702(10)	4.5011(12)		
Cg2	[1]	->	Cg1	[4564.01]	5.0454(15)	0.0211	0.1851	0.9825	2.5195	73.72(12)	45.1	28.6	4.4307(10)	3.5586(12)		
				Min or Max	3.935					0.0	28.6	89.2	-3.428	-4.501		

[3576] = -X,2-Y,1-Z
[4565] = X,3/2-Y,1/2+Z
[3566] = -X,1-Y,1-Z
[4564] = X,3/2-Y,-1/2+Z

Table S3 (cont.). π,π-stacking interactions analysis of compounds MTF—MEF, MTF—TLP and MTF—FLP.

MTF—FLP

6-Membered Ring (1) C5 --> C6 --> C7 --> C8 --> C9 --> C10 -->
6-Membered Ring (2) C11 --> C12 --> C13 --> C14 --> C15 --> C16 -->

Cg(I)	Res(I)	Cg(J)	[ARU(J)]	Cg-Cg	Transformed J-Plane P, Q, R, S	Alpha	Beta	Gamma	CgI_Perp	CgJ_Perp	Slippage	
Cg1	[1] ->	Cg1	[2557.01]	4.015(2)	-0.4231-0.2882-0.8590 -10.4389	0.00(16)	17.4	17.4	3.8302(13)	3.8303(13)	1.202	**FLP-FLP**
Cg1	[1] ->	Cg2	[2556.01]	4.9080(18)	0.1215-0.7915-0.5990 -1.3000	46.27(14)	11.4	40.0	3.7580(13)	4.8113(11)		
Cg2	[1] ->	Cg1	[2556.01]	4.9079(18)	-0.4231-0.2882-0.8590 -1.9151	46.27(14)	40.0	11.4	4.8113(11)	3.7579(13)		
			Min or Max	4.015		0.0	11.4	40.0	-4.811	-4.811		

[2557] = -X,-Y,2-Z
[2556] = -X,-Y,1-Z
